# From hundreds to thousands: Widening the normal human Urinome

**DOI:** 10.1016/j.dib.2014.08.006

**Published:** 2014-08-22

**Authors:** Laura Santucci, Giovanni Candiano, Andrea Petretto, Maurizio Bruschi, Chiara Lavarello, Elvira Inglese, Pier Giorgio Righetti, Gian Marco Ghiggeri

**Affiliations:** a Nephrology, Dialysis, Transplantation Unit and Laboratory on Pathophysiology of Uremia, Istituto Giannina Gaslini, 16148 Genova, Italy; b Laboratory of Mass Spectrometry – Core Facility, Istituto Giannina Gaslini, 16148 Genova, Italy; c Politecnico di Milano, Department of Chemistry, Materials and Chemical Engineering “Giulio Natta”, Via Mancinelli 7, 20131 Milano, Italy

**Keywords:** Urinary proteome, Vesicles, Combinatorial peptide ligand libraries, Mass spectrometry

## Abstract

The limits on protein detection in urine are unknown. Improving the analytical approach to detection would increase the number of identified proteins and potentially strengthen their predictive potential in diseases.

Here, we present the data that resulted from a combination of analytical procedures for maximizing sensitivity and reproducibility of normal human urinary proteome analysis. These procedures are ultracentrifugation, vesicle separation, combinatorial peptide ligand libraries (CPLL) and solvent removal of pigments. Proteins were identified by an Orbitrap Velos Mass Spectrometry. 3429 proteins are characterized, 1724 of which are novel discoveries.

The data are related to Santucci et al. (in press) [1] and available both here and at *ChorusProject.org* under project name “From hundreds to thousands: widening the normal human Urinome”. The material supplied to Chorus Progect.org includes technical MS spectra data only.

**Specifications table**

**Table:** 

Subject area	Biology
More specific subject area	Characterization of urine proteome in normal conditions. It was developed a new strategy of analysis which allowed the extension of the number of identified proteins to 3429 (1724 were of new description here). The list of identified proteins is reported in Supplementary Table 1.
Type of data	Excel tables
How data was acquired	*Ultracentrifuge*: Beckman Optima^TM^ l-90K (Ti 90 rotor).
	*Mass spectroscopy*: for analysis of all urinary sub-fractions was used a linear Trap Quadropole (LTQ) Orbitrap Velos Pro Mass Spectrometry.
Data format	Raw MS files as processed with the Thermo Scientific Proteome Discoverer software: peak were searched by the MASCOT and SEQUEST against Uniprot human database, filtered for a maximum 1% FDR using Percolator; the Peptide Mass Deviation was set to 10 ppm and a minimum of six 6 amino acids per identified peptide were required [2,3]. The Database search parameters were mass tolerance precursor 20 ppm [4], mass tolerance fragment CID 0.8 Da with dynamic modification of deamidation (N, Q), oxidation (M) and static modification of alkylation with IAM (C).
	Label free experiments and statistical analysis were performed with Perseus software.
Experimental factors	*Sample Collection and Urine Preparation*: Second morning urine (approximately 160 mL) from healthy volunteers were collected, centrifuged at 4 °C for 10 min at 1000*g*. Urine were frozen and maintained at −80 until further processing [5–14]. Second step was centrifugation at 17,000*g* for 30 min at 16 °C. The sample, after Bradford protein assay [15], was stored at −80 °C until use.
Experimental features	Procedure used for analysis of the urinary proteome: Vesicle Isolation, butanol precipitation, Combinatorial Solid-Phase Ligand Library chromatography, Mass spectrometry and Bioinformatic analysis.
Consent	Informed consent was obtained from all the participants in the study.
Data source location	Genova, Italy.
Data accessibility	The data are available at *ChorusProject.org* under project name “From hundreds to thousands: widening the normal human Urinome”. Row data relative to MS spectra are available at *ChorusProject.org* under project name “From hundreds to thousands: widening the normal human Urinome”. Data are also directly available with this article

**Value of the data**

• Sub-fractionating normal urine by successive steps allowed to identify 3429 proteins, a net +50% increment compared to traditional methods of analysis.
• Vesicles separation, CPLL and solvent treatments are the basic steps.
• 1724 of the urine proteins identified here are of newly identified and described. Improved characterization of the normal urinary proteome opens doors for the analysis of urine biomarkers in human diseases.

## 1 Data, experimental design, materials and methods

3429 Non-redundant proteins discovered in our urine proteomic analysis are characterized and are noted in Supplementary Table 1 [1]. 1615 of these proteins were contained in vesicles while the remaining 1794 were equally distributed among CPLL (1488) and butanol insoluble fractions (322). Several proteins were detected exclusively in one of the phases of the procedure, suggesting that each step is crucial in the fractionation strategy. Many (1724) proteins are here described whose presence in urine have never been reported and represent a potential source of information considering that urine is the unique site of excretion of products of interaction of metabolic processes.

### 1.1 Urinary vesicles isolation

The 17,000*g* urinary supernatant (80 mL) was ultracentrifuged at 48,000 rpm for 75 min at 18 °C. The ultracentrifugation step was repeated by adding the same volume used before until vesicles [16] were isolated. The pellets, washed in DTT (200 mg/mL) and Tris–HCl 65 mM pH 8.8, was centrifuged a 14,000 rpm for 10 min at 4 °C and stored at −80 °C until mass spectrometry analysis.

### 1.2 Butanol precipitation

The 48,000 rpm supernatant was dialyzed versus water; aliquots of 50 mL were added with 100 µL of acetic acid (about pH 3–4) and 10 mL of n-butanol and centrifuged at 4,000 rpm for 10 min at 18 °C. Three different phases were obtained: protein pellet, supernatant (for ProteoMiner^TM^, see below) and pigments (discarted), yielding two fractions: CPLL-beads chromatography and unbound.

#### 1.2.1 Combinatorial peptide ligand library (CPLL)

The phase deriving from butanol extraction was lyophilized and loaded onto a column of 150 µL peptide library beads equilibrated in 25 mM phosphate buffer, pH 7.4 as already described by Candiano et al. [17,18]. The eluate and the unbound fraction were preserved at −80 °C until analysis by mass spectrometry.

#### 1.2.2 Mass spectrometry

Samples for mass spectrometry were solubilized in 0.1 mL of 4% SDS, 50 mM DTT, and 0.1 M Tris/HCl, pH 7.6, at 90 °C for 5 min and briefly sonicated and were processed by the FASP procedure using 30k Vivacom filtration devices (Sartorius) [19].

The mass spectrometer LTQ-Orbitrap Velos Pro was operated in positive ionization mode.

Single MS survey scans were performed in the Orbitrap, recording a mass window between 350 and 1650 m/z using a maximal ion injection time of 250 ms. The resolution was set to 60,000 and the automatic gain control was set to 1,000,000 ions. The experiments were done in data-dependent acquisition mode with alternating MS and MS/MS experiments. A maximum of 10 MS/MS experiments were triggered per MS scan.

Raw MS files were processed with Thermo Scientific Proteome Discoverer software version 1.3. Peak list files were searched by the MASCOT and SEQUEST search engine against Uniprot human database (Release 2012_07) containing both forward and reversed protein sequences. The Database search parameters are mass tolerance precursor 20 ppm, mass tolerance fragment CID 0.8 Da and a dynamic modification of deamidation (N, Q), oxidation (M). For all searches the option trypsin with two missed cleavages was selected. Proteins were grouped by applying the maximum parsimony rule. Resulting peptide hits were filtered for a maximum 1% FDR using Percolator and a Peptide Mass Deviation of 10 ppm per identified peptide were required. To determine the area for any identified peptides, we used precursor ions area detector node of Proteome Discoverer.

#### 1.2.3 Bioinformatic analysis

All the statistical analyses of the identified protein tables were done with the Perseus program (J. Cox, Department of Proteomics and Signal Transduction, Max Planck Institute of Biochemistry, Martinsried, Germany). For hierarchical clustering, we filtered the data according to the technical replicate and kept proteins with a minimum of 3 values repeated in the different analysis. Logarithmized areas were *z*-scored and clustered using Euclidean distances between averages. Fisher exact tests were done with a Benjamini–Hochberg FDR threshold of 0.02.

## Conflict of interest

All authors declare no conflicts of interest.

## Footnotes

Appendix A Supplementary data associated with this article can be found in the online version at doi:10.1016/j.dib.2014.08.006.
